# The genome sequence of the Mauritius parakeet, *Alexandrinus eques *(formerly *Psittacula eques*) (A.Newton & E. Newton, 1876)

**DOI:** 10.12688/wellcomeopenres.22583.1

**Published:** 2024-07-16

**Authors:** Hernán E. Morales, Jim J. Groombridge, Simon Tollington, Sion Henshaw, Vikash Tatayah, Kevin Ruhomaun, Cock van Oosterhout, M. Thomas P. Gilbert, Wellcome Sanger Institute Tree of Life Management, Samples and Laboratory team, Wellcome Sanger Institute Scientific Operations: Sequencing Operations, Wellcome Sanger Institute Tree of Life Core Informatics team, Tree of Life Core Informatics collective

**Affiliations:** 1 Globe Institute, University of Copenhagen, Copenhagen, Denmark; 2 Centre for Evolutionary Hologenomics, University of Copenhagen, Copenhagen, Denmark; 3 Durrell Institute of Conservation and Ecology, Division of Human and Social Sciences, University of Kent, Canterbury, England, UK; 4 School of Animal, Rural and Environmental Sciences, Nottingham Trent University, Nottingham, England, UK; 5 Mauritian Wildlife Foundation, Vacoas, Mauritius; 6 National Parks and Conservation Service (Government of Mauritius), Reduit, Mauritius; 7 School of Environmental Sciences, University of East Anglia, Norwich, England, UK

**Keywords:** Psittacula eques, Psittacula echo, Alexandrinus eques, Mauritius parakeet, genome sequence, chromosomal, Psittaciformes

## Abstract

We present a genome assembly from an individual male *Alexandrinus eques*, formerly *Psittacula eques* (the Mauritius Parakeet; Chordata; Aves; Psittaciformes; Psittacidae). The genome sequence is 1203.8 megabases in span. Most of the assembly is scaffolded into 35 chromosomal pseudomolecules, including the Z sex chromosome. The mitochondrial genome has also been assembled and is 18.86 kilobases in length.

## Species taxonomy

Eukaryota; Metazoa; Chordata; Aves; Psittaciformes; Psittacidae; Psittacula (NCBI:txid1560315)

## Background

**Figure 1.**
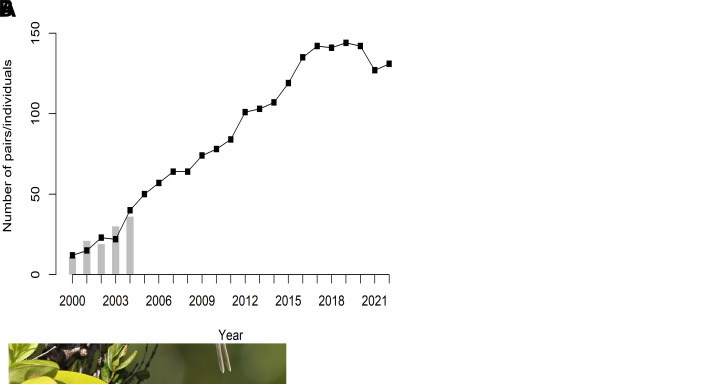
The fall and rise of the Mauritius Parakeet. ( **A**) A male Mauritius Parakeet ( *Alexandrinus eques*; formerly *Psittacula eques;* photo credit Jacques de Speville) ( **B**) Demographic trajectory over time (bottleneck and recovery), the line represents the number of known breeding pairs from the monitoring programme. Total population census sizes are estimated to include non-breeding individuals and sub-adults. The bars represent the number of captive-breed individuals released into the free-living population.

The Mauritius Parakeet ( *Alexandrinus eques*; formerly *Psittacula eques*) is the only surviving endemic species of parrot in Mauritius and the Mascarenes. Characterised by its bright green plumage and red and black markings around the beak and eyes ( Figure 1A), this parakeet feeds predominantly on the fruits, flowers, and leaves of native forest plants. Mauritius Parakeets are known for nesting in natural cavities within mature trees, where they lay between two to four eggs each breeding season, occurring mainly from September to January.

The population of the Mauritius Parakeet plummeted due to habitat loss and invasive species, dwindling to just 20 birds by 1986 and facing imminent extinction ( Jones, 1987; Tatayah *et al.*, 2007). Through three decades of dedicated conservation efforts, including captive-breeding, reintroductions, nest management, predator control, supplementary feeding, and habitat restoration, the population rebounded ( Jones, 2010; Jones *et al.*, 2013; Jones *et al.*, 1998) ( Figure 1B). By 2019, it reached an estimated 750 birds, leading to its downlisting in the IUCN Red List from Critically Endangered to Vulnerable ( BirdLife International, 2019).

In 2005, Psittacine Beak and Feather Disease (PBFD) was detected in the population, a condition characterised by feather dystrophy and immunosuppression, of which the causative agent, Beak and Feather Disease Virus, is one of the most common infections of parrots ( Kundu *et al.*, 2012; Ritchie *et al.*, 1989). Although PBFD can be fatal, with juveniles being particularly susceptible ( Todd, 2000), the species continued to recover despite significant sub-lethal effects detected in the free-living population ( Tollington *et al.*, 2015).

During the decline and recovery of the species, there was a significant loss in genetic diversity, including reduced heterozygosity and allelic richness at microsatellite loci ( Tollington *et al.*, 2013). Initial genetic structure showed differentiation between subpopulations, which has diminished as their size and range expanded due to intensive conservation efforts ( Raisin *et al.*, 2012; Tollington *et al.*, 2013). The ongoing conservation management includes supplementary feeding, which boosts reproductive fitness but may increase BFDV transmission ( Fogell *et al.*, 2019; Fogell *et al.*, 2021; Tollington *et al.*, 2019). Continuous monitoring of genetic diversity, viral prevalence, productivity, and population viability is in place. A vast archive of biological samples and decades of fitness data position the Mauritius Parakeet as an ideal model for studying genomic changes during population recovery and during outbreaks of emergent infectious diseases (EID).

Currently, hundreds of whole genomes are being re-sequenced from historical (pre-1900), recent (1990–2000) and contemporary samples to address these questions. This research efforts are part of a collaboration between several universities (UK - University of Kent, University of East Anglia, Denmark - The University of Copenhagen) with the Government of Mauritius’ National Parks and Conservation Service (NPCS) and the Mauritian Wildlife Foundation (MWF – conservation NGO, Mauritius). The conservation monitoring and management of the Mauritius Parakeet is led by the MWF in collaboration with the NPCS under guidance from the university partners. Recent conservation actions have also been implemented by Ebony Forest Reserve (conservation group).

## Genome sequence report

The genome was sequenced from a blood sample from a male *Alexandrinus eques* collected from Black River Gorges, Mauritius (–20.39, 57.45). A total of 79-fold coverage in Pacific Biosciences single-molecule HiFi long reads was generated. Primary assembly contigs were scaffolded with chromosome conformation Hi-C data. Manual assembly curation corrected 41 missing joins or mis-joins, reducing the scaffold number by 13.13%, and increasing the scaffold N50 by 1.02%.

**Figure 2.**
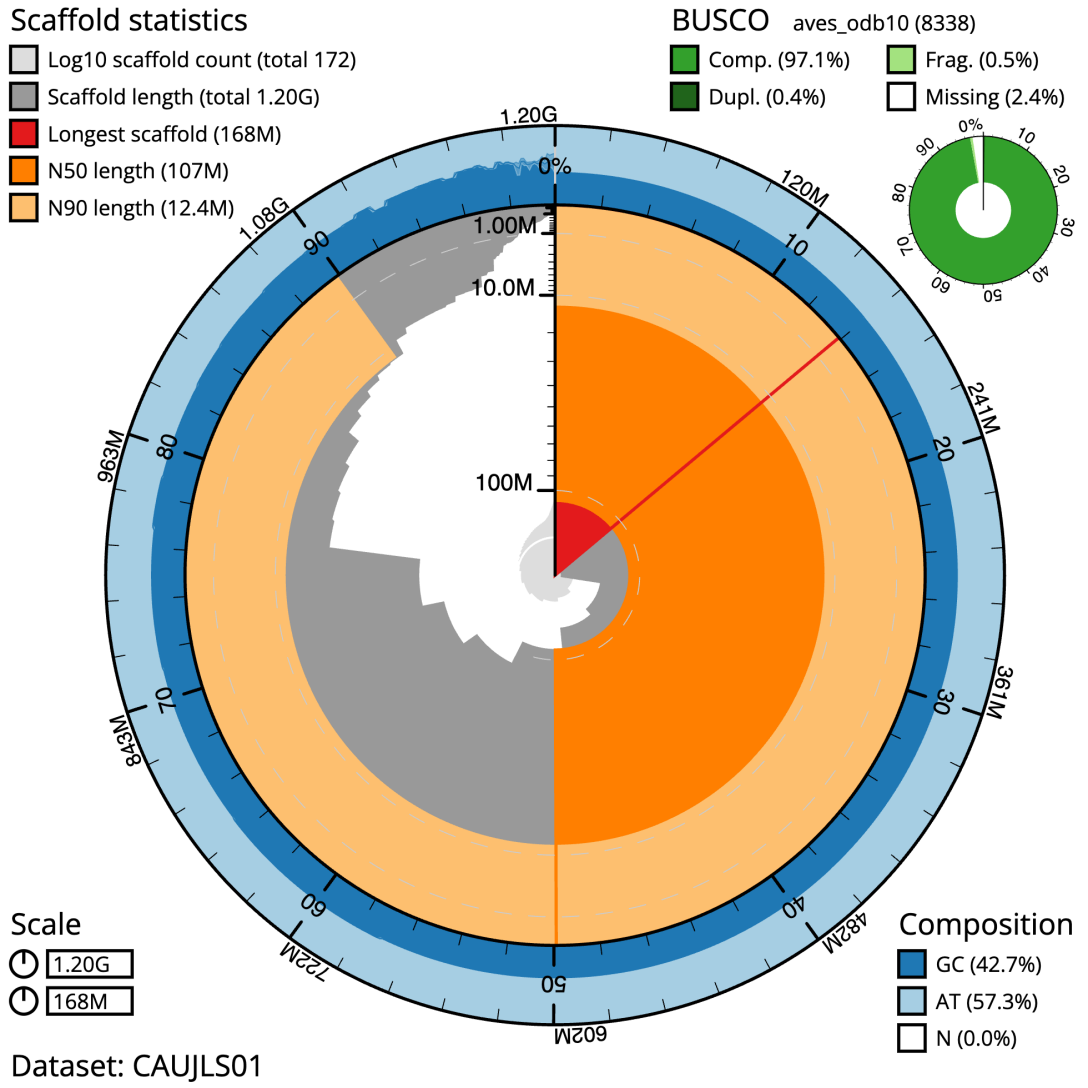
Genome assembly of *Alexandrinus eques* metrics. The BlobToolKit snail plot shows N50 metrics and BUSCO gene completeness. The main plot is divided into 1,000 size-ordered bins around the circumference with each bin representing 0.1% of the 1,203,831,919 bp assembly. The distribution of scaffold lengths is shown in dark grey with the plot radius scaled to the longest scaffold present in the assembly (168,074,981 bp, shown in red). Orange and pale-orange arcs show the N50 and N90 scaffold lengths (106,987,384 and 12,426,226 bp), respectively. The pale grey spiral shows the cumulative scaffold count on a log scale with white scale lines showing successive orders of magnitude. The blue and pale-blue area around the outside of the plot shows the distribution of GC, AT and N percentages in the same bins as the inner plot. A summary of complete, fragmented, duplicated and missing BUSCO genes in the aves_odb10 set is shown in the top right. An interactive version of this figure is available at https://blobtoolkit.genomehubs.org/view/Psittacula%20echo/dataset/CAUJLS01/snail.

**Figure 3.**
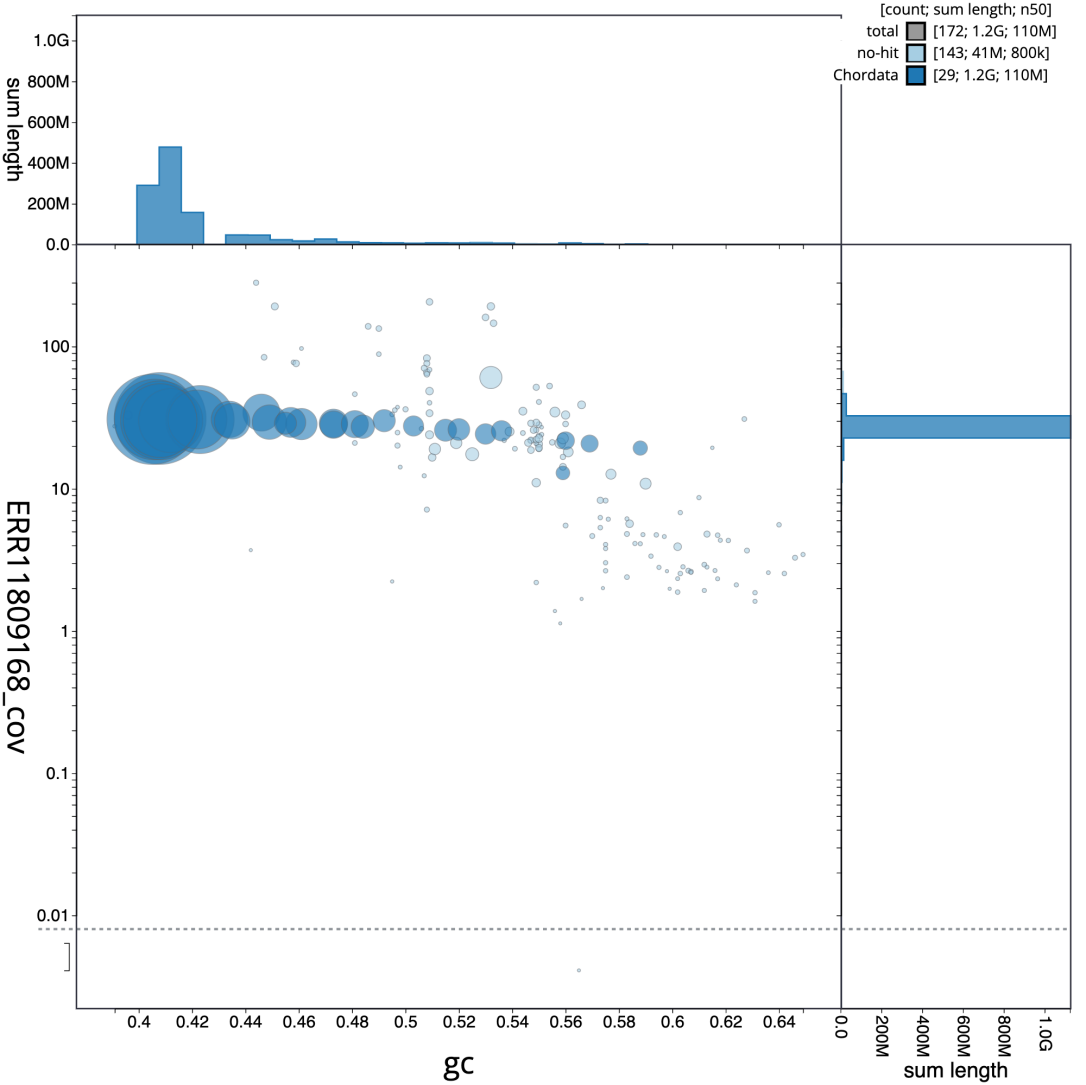
Genome assembly of *Alexandrinus eques*; formerly *Psittacula eques*, bPsiEch3.1: BlobToolKit GC-coverage plot. Sequences are coloured by phylum. Circles are sized in proportion to sequence length. Histograms show the distribution of sequence length sum along each axis. An interactive version of this figure is available at https://blobtoolkit.genomehubs.org/view/Psittacula%20echo/dataset/CAUJLS01/blob.

**Figure 4.**
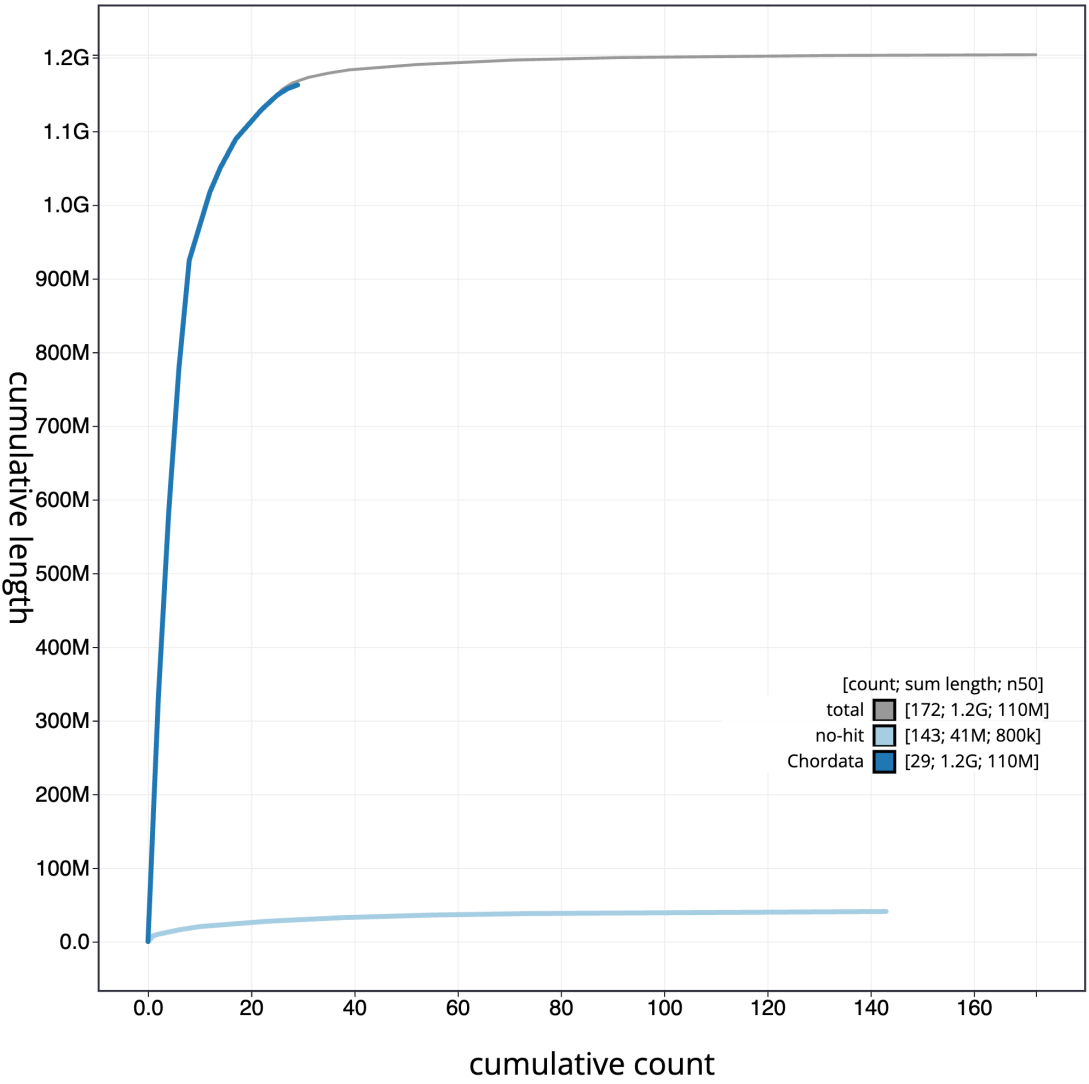
Genome assembly of *Alexandrinus eques*; formerly *Psittacula eques*, bPsiEch3.1: BlobToolKit cumulative sequence plot. The grey line shows cumulative length for all sequences. Coloured lines show cumulative lengths of sequences assigned to each phylum using the buscogenes taxrule. An interactive version of this figure is available at https://blobtoolkit.genomehubs.org/view/Psittacula%20echo/dataset/CAUJLS01/cumulative.

**Figure 5.**
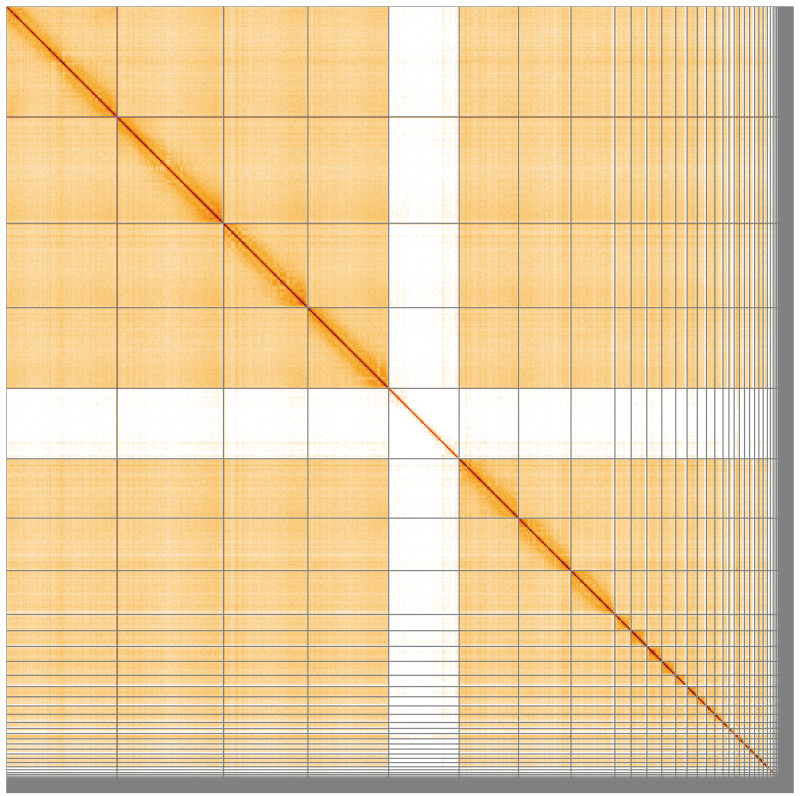
Genome assembly of *Alexandrinus eques*; formerly *Psittacula eques*, bPsiEch3.1: Hi-C contact map of the bPsiEch3.1 assembly, visualised using HiGlass. Chromosomes are shown in order of size from left to right and top to bottom. An interactive version of this figure may be viewed at https://genome-note-higlass.tol.sanger.ac.uk/l/?d=fHq_ahaYS1eLkq54w6IhGA.

The final assembly has a total length of 1203.8 Mb in 171 sequence scaffolds with a scaffold N50 of 107.0 Mb ( Table 1). The snail plot in Figure 2 provides a summary of the assembly statistics, while the distribution of assembly scaffolds on GC proportion and coverage is shown in Figure 3. The cumulative assembly plot in Figure 4 shows curves for subsets of scaffolds assigned to different phyla. Most (97.61%) of the assembly sequence was assigned to 35 chromosomal-level scaffolds, representing 34 autosomes and the Z sex chromosome. Chromosome-scale scaffolds confirmed by the Hi-C data are named in order of size ( Figure 5; Table 2). The Z chromosome was identified based on the Hi-C signal from female sample (Pacbio HiFi data used for *de novo* assembly was derived from a male). While not fully phased, the assembly deposited is of one haplotype. Contigs corresponding to the second haplotype have also been deposited. The mitochondrial genome was also assembled and can be found as a contig within the multifasta file of the genome submission.

**Table 1.** Genome data for *Alexandrinus eques*; formerly *Psittacula eques*, bPsiEch3.1.

Project accession data		
Assembly identifier	bPsiEch3.1	
Species	*Psittacula eques*	
Specimen	bPsiEch3	
NCBI taxonomy ID	232653	
BioProject	PRJEB64768	
BioSample ID	SAMEA12361725	
Isolate information	bPsiEch3, male (PacBio DNA and Illumina RNA sequencing) bPsiEch1, female (Illumina Hi-C sequencing)	
Assembly metrics *		*Benchmark*
Consensus quality (QV)	65.6	*≥ 50*
*k*-mer completeness	100.0%	*≥ 95%*
BUSCO **	C:97.1%[S:96.8%,D:0.3%],F:0.5%, M:2.4%,n:8,338	*C ≥ 95%*
Percentage of assembly mapped to chromosomes	97.61%	*≥ 95%*
Sex chromosomes	Z	*localised homologous pairs*
Organelles	Mitochondrial genome: 18.86 kb	*complete single alleles*
Raw data accessions		
PacificBiosciences Sequel IIe, Revio	ERR11809169, ERR11809168	
Hi-C Illumina	ERR11814144	
PolyA RNA-Seq Illumina	ERR11814145	
Genome assembly		
Assembly accession	GCA_963264785.1	
*Accession of alternate haplotype*	GCA_963243765.1	
Span (Mb)	1203.8	
Number of contigs	530	
Contig N50 length (Mb)	5.9	
Number of scaffolds	171	
Scaffold N50 length (Mb)	107.0	
Longest scaffold (Mb)	168.07	

* Assembly metric benchmarks are adapted from column VGP-2020 of “Table 1: Proposed standards and metrics for defining genome assembly quality” from Rhie *et al.* (2021). ** BUSCO scores based on the vertebrata_odb10 BUSCO set using version v5.4.3. C = complete [S = single copy, D = duplicated], F = fragmented, M = missing, n = number of orthologues in comparison. A full set of BUSCO scores is available at https://blobtoolkit.genomehubs.org/view/Psittacula%20echo/dataset/CAUJLS01/busco.

**Table 2.** Chromosomal pseudomolecules in the genome assembly of *Alexandrinus eques*; formerly *Psittacula eques*, bPsiEch3.

INSDC accession	Chromosome	Length (Mb)	GC%
OY725420.1	1	168.07	41.0
OY725421.1	2	161.94	40.5
OY725422.1	3	128.36	40.5
OY725423.1	4	122.79	41.0
OY725425.1	5	90.32	42.5
OY725426.1	6	79.82	41.0
OY725427.1	7	66.74	42.0
OY725428.1	8	24.68	44.5
OY725429.1	9	23.83	43.5
OY725430.1	10	22.8	43.5
OY725431.1	11	21.18	45.0
OY725432.1	12	17.15	46.0
OY725433.1	13	15.5	45.5
OY725434.1	14	13.94	47.5
OY725435.1	15	12.76	47.5
OY725436.1	16	12.43	48.0
OY725437.1	17	8.98	48.5
OY725438.1	18	7.91	49.0
OY725439.1	19	7.9	53.0
OY725440.1	20	7.85	45.5
OY725441.1	21	7.78	51.5
OY725442.1	22	7.5	52.0
OY725443.1	23	6.57	53.0
OY725444.1	24	6.49	53.5
OY725445.1	25	6.37	50.5
OY725446.1	26	4.64	56.0
OY725447.1	27	4.27	57.0
OY725448.1	28	2.76	59.0
OY725449.1	29	2.47	56.0
OY725450.1	30	1.41	59.0
OY725451.1	31	1.11	57.5
OY725452.1	32	0.49	60.5
OY725453.1	33	0.48	58.5
OY725454.1	34	0.23	61.5
OY725424.1	Z	106.99	41.0
OY725455.1	MT	0.02	46.0

The estimated Quality Value (QV) of the final assembly is 65.6 with *k*-mer completeness of 100.0%, and the assembly has a BUSCO v5.4.3 completeness of 97.1% (single = 96.8%, duplicated = 0.3%), using the vertebrata_odb10 reference set ( *n* = 8,338).

Metadata for specimens, BOLD barcode results, spectra estimates, sequencing runs, contaminants and pre-curation assembly statistics are given at https://links.tol.sanger.ac.uk/species/232653.

## Methods

### Sample acquisition and nucleic acid extraction

Blood sampling of Mauritius Parakeets is routinely conducted by the MWF and university researchers, overseen by the International Zoo Veterinary Group (IZVG). Samples are taken from the jugular and brachial veins of chicks at 45 days old in their nests and from adults captured at nest sites or field aviaries during health checks. Extensive blood sampling has been performed throughout the species’ restoration, with most chicks and some adults sampled opportunistically. For genomic sequencing, blood from select individuals was preserved in absolute ethanol and immediately frozen to minimize DNA degradation. The genome and RNA was sequenced from a male bird (specimen ID SAN1100033, ToLID bPsiEch3) caught in the field aviary on 2020-01-15, while a female specimen (specimen ID SAN1100031, ToLID bPsiEch1) collected on 2020-01-29 was used for Hi-C sequencing.

The workflow for high molecular weight (HMW) DNA extraction at the Wellcome Sanger Institute (WSI) Tree of Life Core Laboratory includes a sequence of core procedures: sample preparation; sample homogenisation, DNA extraction, fragmentation, and clean-up. In sample preparation, the bPsiEch3 sample was weighed and kept on dry ice ( Jay *et al.*, 2023). For sample homogenisation, blood was cryogenically disrupted using the Covaris cryoPREP ^®^ Automated Dry Pulverizer ( Narváez-Gómez *et al.*, 2023). HMW DNA was extracted using the manual Nucleated Blood Nanobind ^®^ protocol ( Denton *et al.*, 2023a). DNA was sheared into an average fragment size of 12–20 kb in a Megaruptor 3 system with speed setting 31 ( Bates *et al.*, 2023). Sheared DNA was purified by solid-phase reversible immobilisation ( Strickland *et al.*, 2023). The concentration of the sheared and purified DNA was assessed using a Nanodrop spectrophotometer and Qubit Fluorometer and Qubit dsDNA High Sensitivity Assay kit. Fragment size distribution was evaluated by running the sample on the FemtoPulse system.

RNA was extracted from the bPsiEch3 blood sample in the Tree of Life Laboratory at the WSI using the RNA Extraction: Automated MagMax™ *mir*Vana protocol ( do Amaral *et al.*, 2023). The RNA concentration was assessed using a Nanodrop spectrophotometer and a Qubit Fluorometer using the Qubit RNA Broad-Range Assay kit. Analysis of the integrity of the RNA was done using the Agilent RNA 6000 Pico Kit and Eukaryotic Total RNA assay.

Protocols developed by the WSI Tree of Life laboratory are publicly available on protocols.io ( Denton *et al.*, 2023b).

### Sequencing

Pacific Biosciences HiFi circular consensus DNA sequencing libraries were constructed according to the manufacturers’ instructions. Poly(A) RNA-Seq libraries were constructed using the NEB Ultra II RNA Library Prep kit. DNA and RNA sequencing was performed by the Scientific Operations core at the WSI on Pacific Biosciences Sequel IIe, Revio (HiFi) and Illumina NovaSeq 6000 (RNA-Seq) instruments. Hi-C data were also generated from blood from bPsiEch1 using the Arima2 kit and sequenced on the Illumina NovaSeq 6000 instrument.

### Genome assembly and curation

Assembly was carried out with Hifiasm ( Cheng *et al.*, 2021) and haplotypic duplication was identified and removed with purge_dups ( Guan *et al.*, 2020). The assembly was then scaffolded with Hi-C data ( Rao *et al.*, 2014) using YaHS ( Zhou *et al.*, 2023). The assembly was checked for contamination and corrected using the TreeVal pipeline ( Pointon *et al.*, 2023). Manual curation was performed using JBrowse2 ( Diesh *et al.*, 2023), HiGlass ( Kerpedjiev *et al.*, 2018) and PretextView ( Harry, 2022). The mitochondrial genome was assembled using MitoHiFi ( Uliano-Silva *et al.*, 2023), which runs MitoFinder ( Allio *et al.*, 2020) or MITOS ( Bernt *et al.*, 2013) and uses these annotations to select the final mitochondrial contig and to ensure the general quality of the sequence.

### Final assembly evaluation

The final assembly was post-processed and evaluated with the three Nextflow ( Di Tommaso *et al.*, 2017) DSL2 pipelines “sanger-tol/readmapping” ( Surana *et al.*, 2023a), “sanger-tol/genomenote” ( Surana *et al.*, 2023b), and “sanger-tol/blobtoolkit” ( Muffato *et al.*, 2024). The pipeline sanger-tol/readmapping aligns the Hi-C reads with bwa-mem2 ( Vasimuddin *et al.*, 2019) and combines the alignment files with SAMtools ( Danecek *et al.*, 2021). The sanger-tol/genomenote pipeline transforms the Hi-C alignments into a contact map with BEDTools ( Quinlan & Hall, 2010) and the Cooler tool suite ( Abdennur & Mirny, 2020), which is then visualised with HiGlass ( Kerpedjiev *et al.*, 2018). It also provides statistics about the assembly with the NCBI datasets ( Sayers *et al.*, 2024) report, computes *k*-mer completeness and QV consensus quality values with FastK and MerquryFK, and a completeness assessment with BUSCO ( Manni *et al.*, 2021).

The sanger-tol/blobtoolkit pipeline is a Nextflow port of the previous Snakemake Blobtoolkit pipeline ( Challis *et al.*, 2020). It aligns the PacBio reads with SAMtools and minimap2 ( Li, 2018) and generates coverage tracks for regions of fixed size. In parallel, it queries the GoaT database ( Challis *et al.*, 2023) to identify all matching BUSCO lineages to run BUSCO ( Manni *et al.*, 2021). For the three domain-level BUSCO lineage, the pipeline aligns the BUSCO genes to the Uniprot Reference Proteomes database ( Bateman *et al.*, 2023) with DIAMOND ( Buchfink *et al.*, 2021) blastp. The genome is also split into chunks according to the density of the BUSCO genes from the closest taxonomically lineage, and each chunk is aligned to the Uniprot Reference Proteomes database with DIAMOND blastx. Genome sequences that have no hit are then chunked with seqtk and aligned to the NT database with blastn ( Altschul *et al.*, 1990). All those outputs are combined with the blobtools suite into a blobdir for visualisation.

All three pipelines were developed using the nf-core tooling ( Ewels *et al.*, 2020), use MultiQC ( Ewels *et al.*, 2016), and make extensive use of the Conda package manager, the Bioconda initiative ( Grüning *et al.*, 2018), the Biocontainers infrastructure ( da Veiga Leprevost *et al.*, 2017), and the Docker ( Merkel, 2014) and Singularity ( Kurtzer *et al.*, 2017) containerisation solutions.

Table 3 contains a list of relevant software tool versions and sources.

**Table 3.** Software tools: versions and sources.

Software tool	Version	Source
BEDTools	2.30.0	https://github.com/arq5x/bedtools2
Blast	2.14.0	ftp://ftp.ncbi.nlm.nih.gov/blast/executables/blast+/
BlobToolKit	4.3.7	https://github.com/blobtoolkit/blobtoolkit
BUSCO	5.4.3	https://gitlab.com/ezlab/busco
BUSCO	5.4.3 and 5.5.0	https://gitlab.com/ezlab/busco
bwa-mem2	2.2.1	https://github.com/bwa-mem2/bwa-mem2
Cooler	0.8.11	https://github.com/open2c/cooler
DIAMOND	2.1.8	https://github.com/bbuchfink/diamond
fasta_windows	0.2.4	https://github.com/tolkit/fasta_windows
FastK	427104ea91c78c3b8b8b49f1a7d6bbeaa869ba1c	https://github.com/thegenemyers/FASTK
GoaT CLI	0.2.5	https://github.com/genomehubs/goat-cli
Hifiasm	0.16.1-r375	https://github.com/chhylp123/hifiasm
HiGlass	1.11.6	https://github.com/higlass/higlass
HiGlass	44086069ee7d4d3f6f3f0012569789ec138f42b84aa44357826c0b6753eb28de	https://github.com/higlass/higlass
MerquryFK	d00d98157618f4e8d1a9190026b19b471055b22e	https://github.com/thegenemyers/MERQURY.FK
MitoHiFi	2	https://github.com/marcelauliano/MitoHiFi
MultiQC	1.14, 1.17, and 1.18	https://github.com/MultiQC/MultiQC
NCBI Datasets	15.12.0	https://github.com/ncbi/datasets
Nextflow	23.04.0-5857	https://github.com/nextflow-io/nextflow
PretextView	0.2	https://github.com/wtsi-hpag/PretextView
purge_dups	1.2.3	https://github.com/dfguan/purge_dups
samtools	1.16.1, 1.17, and 1.18	https://github.com/samtools/samtools
sanger-tol/genomenote	1.1.1	https://github.com/sanger-tol/genomenote
sanger-tol/readmapping	1.2.1	https://github.com/sanger-tol/readmapping
Seqtk	1.3	https://github.com/lh3/seqtk
Singularity	3.9.0	https://github.com/sylabs/singularity
TreeVal	1.0.0	https://github.com/sanger-tol/treeval
YaHS	yahs-1.1.91eebc2	https://github.com/c-zhou/yahs

### Wellcome Sanger Institute – Legal and Governance

The materials that have contributed to this genome note have been supplied by a Tree of Life collaborator. The Wellcome Sanger Institute employs a process whereby due diligence is carried out proportionate to the nature of the materials themselves, and the circumstances under which they have been/are to be collected and provided for use. The purpose of this is to address and mitigate any potential legal and/or ethical implications of receipt and use of the materials as part of the research project, and to ensure that in doing so we align with best practice wherever possible.

The overarching areas of consideration are:

•      Ethical review of provenance and sourcing of the material

•      Legality of collection, transfer and use (national and international)

Each transfer of samples is undertaken according to a Research Collaboration Agreement or Material Transfer Agreement entered into by the Tree of Life collaborator, Genome Research Limited (operating as the Wellcome Sanger Institute) and in some circumstances other Tree of Life collaborators.

## Data Availability

European Nucleotide Archive: *Psittacula echo* (Mauritius parakeet). Accession number PRJEB64768; https://identifiers.org/ena.embl/PRJEB64768 ( Wellcome Sanger Institute, 2023). The genome sequence is released openly for reuse. The *Psittacula eques* genome sequencing initiative is part of the Vertebrate Genomes Project. All raw sequence data and the assembly have been deposited in INSDC databases. The genome will be annotated using available RNA-Seq data and presented through the Ensembl pipeline at the European Bioinformatics Institute. Raw data and assembly accession identifiers are reported in Table 1.

## Author information

Members of the Wellcome Sanger Institute Tree of Life Management, Samples and Laboratory team are listed here: https://doi.org/10.5281/zenodo.10066175.

Members of Wellcome Sanger Institute Scientific Operations: Sequencing Operations are listed here: https://doi.org/10.5281/zenodo.10043364.

Members of the Wellcome Sanger Institute Tree of Life Core Informatics team are listed here: https://doi.org/10.5281/zenodo.10066637.

Members of the Tree of Life Core Informatics collective are listed here: https://doi.org/10.5281/zenodo.5013541.

## References

[ref-1] Abdennur N Mirny LA : Cooler: scalable storage for Hi-C data and other genomically labeled arrays. *Bioinformatics.* 2020;36(1):311–316. 10.1093/bioinformatics/btz540 31290943 PMC8205516

[ref-2] Allio R Schomaker-Bastos A Romiguier J : MitoFinder: efficient automated large-scale extraction of mitogenomic data in target enrichment phylogenomics. *Mol Ecol Resour.* 2020;20(4):892–905. 10.1111/1755-0998.13160 32243090 PMC7497042

[ref-3] Altschul SF Gish W Miller W : Basic local alignment search tool. *J Mol Biol.* 1990;215(3):403–410. 10.1016/S0022-2836(05)80360-2 2231712

[ref-4] Bateman A Martin MJ Orchard S : UniProt: the universal protein knowledgebase in 2023. *Nucleic Acids Res.* 2023;51(D1):D523–D531. 10.1093/nar/gkac1052 36408920 PMC9825514

[ref-5] Bates A Clayton-Lucey I Howard C : Sanger tree of life HMW DNA fragmentation: diagenode Megaruptor ^®^3 for LI PacBio. *protocols.io.* 2023. 10.17504/protocols.io.81wgbxzq3lpk/v1

[ref-6] Bernt M Donath A Jühling F : MITOS: improved *de novo* metazoan mitochondrial genome annotation. *Mol Phylogenet Evol.* 2013;69(2):313–319. 10.1016/j.ympev.2012.08.023 22982435

[ref-7] BirdLife International: Psittacula eques, the IUCN red list of threatened species.2019; [Accessed 25 April 2024]. 10.2305/IUCN.UK.2019-3.RLTS.T22685448A154065622.en

[ref-8] Buchfink B Reuter K Drost HG : Sensitive protein alignments at Tree-of-Life scale using DIAMOND. *Nat Methods * 2021;18(4):366–368. 10.1038/s41592-021-01101-x 33828273 PMC8026399

[ref-9] Challis R Kumar S Sotero-Caio C : Genomes on a Tree (GoaT): a versatile, scalable search engine for genomic and sequencing project metadata across the eukaryotic tree of life [version 1; peer review: 2 approved]. *Wellcome Open Res.* 2023;8:24. 10.12688/wellcomeopenres.18658.1 36864925 PMC9971660

[ref-10] Challis R Richards E Rajan J : BlobToolKit – interactive quality assessment of genome assemblies. *G3 (Bethesda).* 2020;10(4):1361–1374. 10.1534/g3.119.400908 32071071 PMC7144090

[ref-11] Cheng H Concepcion GT Feng X : Haplotype-resolved *de novo* assembly using phased assembly graphs with hifiasm. *Nat Methods.* 2021;18(2):170–175. 10.1038/s41592-020-01056-5 33526886 PMC7961889

[ref-12] Danecek P Bonfield JK Liddle J : Twelve years of SAMtools and BCFtools. *GigaScience.* 2021;10(2): giab008. 10.1093/gigascience/giab008 33590861 PMC7931819

[ref-13] da Veiga Leprevost F Grüning BA Alves Aflitos S : BioContainers: an open-source and community-driven framework for software standardization. *Bioinformatics.* 2017;33(16):2580–2582. 10.1093/bioinformatics/btx192 28379341 PMC5870671

[ref-14] Denton A Oatley G Pacific Biosciences and Howard C : Sanger Tree of Life HMW DNA Extraction: Manual Nucleated Blood Nanobind®. *protocols.io.* 2023a. 10.17504/protocols.io.5jyl8p2w8g2w/v1

[ref-15] Denton A Yatsenko H Jay J : Sanger tree of life wet laboratory protocol collection V.1. *protocols.io.* 2023b. 10.17504/protocols.io.8epv5xxy6g1b/v1

[ref-16] Di Tommaso P Chatzou M Floden EW : Nextflow enables reproducible computational workflows. *Nat Biotechnol.* 2017;35(4):316–319. 10.1038/nbt.3820 28398311

[ref-17] Diesh C Stevens GJ Xie P : JBrowse2: a modular genome browser with views of synteny and structural variation. *Genome Biol.* 2023;24(1): 74. 10.1186/s13059-023-02914-z 37069644 PMC10108523

[ref-18] do Amaral RJV Bates A Denton A : Sanger tree of life RNA extraction: automated MagMax ^TM^ mirVana. *protocols.io.* 2023. 10.17504/protocols.io.6qpvr36n3vmk/v1

[ref-20] Ewels P Magnusson M Lundin S : MultiQC: summarize analysis results for multiple tools and samples in a single report. *Bioinformatics.* 2016;32(19):3047–3048. 10.1093/bioinformatics/btw354 27312411 PMC5039924

[ref-19] Ewels PA Peltzer A Fillinger S : The nf-core framework for community-curated bioinformatics pipelines. *Nat Biotechnol.* 2020;38(3):276–278. 10.1038/s41587-020-0439-x 32055031

[ref-21] Fogell DJ Groombridge JJ Tollington S : Hygiene and biosecurity protocols reduce infection prevalence but do not improve fledging success in an endangered parrot. *Sci Rep.* 2019;9(1): 4779. 10.1038/s41598-019-41323-w 30886308 PMC6423005

[ref-22] Fogell DJ Tollington S Tatayah V : Evolution of beak and feather disease virus across three decades of conservation intervention for population recovery of the mauritius parakeet. *Diversity.* 2021;13(11):584. 10.3390/d13110584

[ref-23] Grüning B Dale R Sjödin A : Bioconda: sustainable and comprehensive software distribution for the life sciences. *Nat Methods.* 2018;15(7):475–476. 10.1038/s41592-018-0046-7 29967506 PMC11070151

[ref-24] Guan D McCarthy SA Wood J : Identifying and removing haplotypic duplication in primary genome assemblies. *Bioinformatics.* 2020;36(9):2896–2898. 10.1093/bioinformatics/btaa025 31971576 PMC7203741

[ref-25] Harry E : PretextView (Paired Read Texture Viewer): a desktop application for viewing pretext contact maps. 2022; [Accessed 19 October 2022]. Reference Source

[ref-26] Jay J Yatsenko H Narváez-Gómez JP : Sanger Tree of Life sample preparation: triage and dissection. *protocols.io.* 2023. 10.17504/protocols.io.x54v9prmqg3e/v1

[ref-27] Jones CG : The larger land birds of mauritius. In: Diamond, A. W. (ed.) *Studies of Mascarene Island Birds*. London, UK: Cambridge University Press,1987. 10.1017/CBO9780511735769.007

[ref-28] Jones CG : Back from the brink: the echo parakeet story. *PsittaScene.* 2010;22(3):3–5.

[ref-29] Jones CG M J R A : Echo parakeet psittacula eques. In: Safford, R. J. and Hawkins, A. F. A. (eds.) *The Birds of Africa. Vol. VIII: The Malagasy Region.*London: Christopher Helm,2013;8:517–522.

[ref-30] Jones CG Swinnerton K Thorsen M : The biology and conservation of the echo parakeet psittacula eques of mauritius. In: *Proceedings of the IVth International Parrot Convention*.1998;110–123.

[ref-31] Kerpedjiev P Abdennur N Lekschas F : Higlass: web-based visual exploration and analysis of genome interaction maps. *Genome Biol.* 2018;19(1): 125. 10.1186/s13059-018-1486-1 30143029 PMC6109259

[ref-32] Kundu S Faulkes CG Greenwood AG : Tracking viral evolution during a disease outbreak: the rapid and complete selective sweep of a circovirus in the endangered echo parakeet. *J Virol.* 2012;86(9):5221–9. 10.1128/JVI.06504-11 22345474 PMC3347377

[ref-33] Kurtzer GM Sochat V Bauer MW : Singularity: scientific containers for mobility of compute. *PLoS One.* 2017;12(5): e0177459. 10.1371/journal.pone.0177459 28494014 PMC5426675

[ref-34] Li H : Minimap2: pairwise alignment for nucleotide sequences. *Bioinformatics.* 2018;34(18):3094–3100. 10.1093/bioinformatics/bty191 29750242 PMC6137996

[ref-35] Manni M Berkeley MR Seppey M : BUSCO update: novel and streamlined workflows along with broader and deeper phylogenetic coverage for scoring of eukaryotic, prokaryotic, and viral genomes. *Mol Biol Evol.* 2021;38(10):4647–4654. 10.1093/molbev/msab199 34320186 PMC8476166

[ref-36] Merkel D : Docker: lightweight linux containers for consistent development and deployment. *Linux J.* 2014;2014(239): 2. 10.5555/2600239.2600241

[ref-37] Muffato M Butt Z Challis R : Sanger-tol/blobtoolkit: v0.3.0 - poliwag.2024. 10.5281/zenodo.10649272

[ref-38] Narváez-Gómez JP Mbye H Oatley G : Sanger tree of life sample homogenisation: covaris cryoPREP® automated dry pulverizer V.1. *protocols.io.* 2023. 10.17504/protocols.io.eq2lyjp5qlx9/v1

[ref-39] Pointon DL Eagles W Sims Y : sanger-tol/treeval v1.0.0 - ancient atlantis.2023. 10.5281/zenodo.10047654

[ref-40] Quinlan AR Hall IM : BEDTools: a flexible suite of utilities for comparing genomic features. *Bioinformatics.* 2010;26(6):841–842. 10.1093/bioinformatics/btq033 20110278 PMC2832824

[ref-41] Raisin C Frantz AC Kundu S : Genetic consequences of intensive conservation management for the mauritius parakeet. *Conserv Genet.* 2012;13(3):707–715. 10.1007/s10592-012-0319-0

[ref-42] Rao SSP Huntley MH Durand NC : A 3D map of the human genome at kilobase resolution reveals principles of chromatin looping. *Cell.* 2014;159(7):1665–1680. 10.1016/j.cell.2014.11.021 25497547 PMC5635824

[ref-43] Ritchie BW Niagro FD Lukert PD : A review of psittacine beak and feather disease: characteristics of the PBFD virus. *J Assoc Avian Vet.* 1989;3(3):143–149. 10.2307/30143076

[ref-44] Rhie A McCarthy SA Fedrigo O : Towards complete and error-free genome assemblies of all vertebrate species. *Nature.* 2021;592(7856):737–746. 10.1038/s41586-021-03451-0 33911273 PMC8081667

[ref-45] Sayers EW Cavanaugh M Clark K : GenBank 2024 update. *Nucleic Acids Res.* 2024;52(D1):D134–D137. 10.1093/nar/gkad903 37889039 PMC10767886

[ref-46] Strickland M Cornwell C Howard C : Sanger tree of life fragmented DNA clean up: manual SPRI. *protocols.io.* 2023. 10.17504/protocols.io.kxygx3y1dg8j/v1

[ref-47] Surana P Muffato M Qi G : Sanger-tol/readmapping: sanger-tol/readmapping v1.1.0 - hebridean black (1.1.0). *Zenodo.* 2023a. 10.5281/zenodo.7755669

[ref-48] Surana P Muffato M Sadasivan Baby C : Sanger-tol/genomenote (v1.0.dev). *Zenodo.* 2023b. 10.5281/zenodo.6785935

[ref-49] Tatayah RVV Malham J Haverson J : Design and provision of nest boxes for echo parakeets *psittacula eques* in black river gorges national park, mauritius. *Conserv Evid.* 2007;4:16–19. Reference Source

[ref-50] Todd D : Circoviruses: immunosuppressive threats to avian species: a review. *Avian Pathol.* 2000;29(5):373–94. 10.1080/030794500750047126 19184829

[ref-51] Tollington S Ewen JG Newton J : Individual consumption of supplemental food as a predictor of reproductive performance and viral infection intensity. *J Appl Ecol.* 2019;56(3):594–603. 10.1111/1365-2664.13303

[ref-52] Tollington S Greenwood A Jones CG : Detailed monitoring of a small but recovering population reveals sublethal effects of disease and unexpected interactions with supplemental feeding. *J Anim Ecol.* 2015;84(4):969–977. 10.1111/1365-2656.12348 25757031 PMC5098166

[ref-53] Tollington S Jones CG Greenwood A : Long-term, fine-scale temporal patterns of genetic diversity in the restored mauritius parakeet reveal genetic impacts of management and associated demographic effects on reintroduction programmes. *Biol Conserv.* 2013;161:28–38. 10.1016/j.biocon.2013.02.013

[ref-54] Uliano-Silva M Ferreira JGRN Krasheninnikova K : MitoHiFi: a python pipeline for mitochondrial genome assembly from PacBio high fidelity reads. *BMC Bioinformatics.* 2023;24(1): 288. 10.1186/s12859-023-05385-y 37464285 PMC10354987

[ref-55] Vasimuddin M Misra S Li H : Efficient architecture-aware acceleration of BWA-MEM for multicore systems. In: *2019 IEEE International Parallel and Distributed Processing Symposium (IPDPS)*. IEEE,2019;314–324. 10.1109/IPDPS.2019.00041

[ref-56] Wellcome Sanger Institute: The genome sequence of the Mauritius Parakeet, *Alexandrinus eques* (formerly Psittacula eques) (A.Newton & E.Newton, 1876). European Nucleotide Archive, [dataset], accession number PRJEB64768.2023. 10.12688/wellcomeopenres.22583.1 PMC11411241 39301440

[ref-57] Zhou C McCarthy SA Durbin R : YaHS: yet another Hi-C scaffolding tool. *Bioinformatics.* 2023;39(1): btac808. 10.1093/bioinformatics/btac808 36525368 PMC9848053

