# Supplementary material for: The genome sequence of the Mauritius parakeet, Alexandrinus eques (formerly Psittacula eques) (A.Newton & E. Newton, 1876)
Source: Wellcome Open Res. 2024 Jul 16;9:378. [Version 1] doi: 10.12688/wellcomeopenres.22583.1 (PMC11411241; doi:10.12688/wellcomeopenres.22583.1)
Supplement: Peer review history

# Reviewer report: Reviewer response for version 1

**DOI:** 10.21956/wellcomeopenres.24881.r94859

**Published:** 2024-09-18

**Authors:** Jana Wold

**Affiliations:** 1 University of Canterbury, Christchurch, New Zealand

Here, Morales et al provide a high quality genome assembly for a threatened parrot. This assembly will become a foundational genomic resource for a investigation into the genomic diversity of the Mauritius Parakeet. The assembly, and RNA-seq informed annotation, and may be a powerful tool in the continued conservation efforts for this species.

 The methods implemented here are well considered and appropriate. I have full confidence that the assembly will be a useful resource going forward.

Are sufficient details of methods and materials provided to allow replication by others?

Yes

Is the rationale for creating the dataset(s) clearly described?

Yes

Are the datasets clearly presented in a useable and accessible format?

Yes

Are the protocols appropriate and is the work technically sound?

Yes

Reviewer Expertise:

Conservation genomics

I confirm that I have read this submission and believe that I have an appropriate level of expertise to confirm that it is of an acceptable scientific standard.

# Reviewer report: Reviewer response for version 1

**DOI:** 10.21956/wellcomeopenres.24881.r94861

**Published:** 2024-09-05

**Authors:** Jérôme Fuchs

**Affiliations:** 1 Institut de Systématique Evolution Biodiversité, Muséum national d'Histoire naturelle CNRS SU EPHE UA, Paris, France

The following manuscript describes a chromosome level assembly of the endemic Mauritius parakeet Alexandrinus equees, a species that went through a very strong population bottleneck

 Maybe it is worth mentionning that, as currently understood, the Mauritius Parakeet is nested within or very closely related to the Ring-necked Parakeet (A. krameri)

 I have very few comments that the authors may consider.

 "Most of the assembly sequence was assigned to 35 pseudomolecules, representing 34 autosomes and the Z sex chromosome."

 2n= 70 is coherent with what is known for Alexandrinus species (Ray-Chaudhuri et al. 1969), although not identical to its sister species; maybe worth mentioning somewhere in the manuscript

 Note that on Genbank, the genome project is for Psittacula echo, which is the endemic subspecies to Mauritius. It may be worth to homogenize the names between Genbank and the present manuscript, either by using Alexandrinus eques echo throughout the manuscript and mention that the extinct nominate subspecies is found on Reunion Is or change the name on genbank.

 As part of a genome description publication, it may be useful to further comparative analyses have the proportion of:

- The percentage of repeat sequences
- Number of protein coding genes (since RNA was sequenced)

and possibly: 

- Number of snps in the genome and per chromosome

The two former descriptive statistics are routinely mentioned in genome papers

Are sufficient details of methods and materials provided to allow replication by others?

Yes

Is the rationale for creating the dataset(s) clearly described?

Yes

Are the datasets clearly presented in a useable and accessible format?

Yes

Are the protocols appropriate and is the work technically sound?

Yes

Reviewer Expertise:

Molecular Systematics, Genomics

I confirm that I have read this submission and believe that I have an appropriate level of expertise to confirm that it is of an acceptable scientific standard.

## References

[1] Ray-Chaudhuri R, Sharma T, Ray-Chaudhuri SP. A comparative study of the chromosomes of birds. Chromosoma, 26: 148-168, 1969.
